# Building happier bonds: gratitude as a mediator between dyadic coping and relationship satisfaction in romantic couples

**DOI:** 10.3389/fpsyg.2024.1452397

**Published:** 2024-12-13

**Authors:** Michelle Roth, Nicolas Good, Thomas Ledermann, Selina A. Landolt, Katharina Weitkamp, Guy Bodenmann

**Affiliations:** ^1^Clinical Psychology for Children/Adolescents and Couples/Families, Department of Psychology, University of Zurich, Zurich, Switzerland; ^2^Department of Human Development and Family Science, Florida State University, Tallahassee, FL, United States

**Keywords:** romantic relationships, couples, gratitude, dyadic coping, relationship satisfaction, actor partner interdependence mediation model, dyadic analyses

## Abstract

**Introduction:**

Gratitude has been found to be relevant for relational well-being, and there has been ongoing interest in uncovering the mechanisms by which gratitude functions in interpersonal relationships. Only recently, gratitude has been studied within the context of dyadic coping—the interpersonal process of how partners communicate their stress, support each other during stressful times, and jointly cope with stress—in romantic couples. Drawing up on theoretical models on the functions of gratitude within close relationships and previous research, we aimed to advance this line of research and examined the potential mediating role of gratitude between dyadic coping and relationship satisfaction applying a dyadic perspective. For a more nuanced understanding of gratitude, we differentiated between felt and expressed dyadic coping-related gratitude.

**Methods:**

We used data of 163 romantic mixed-gender couples living in Switzerland. To examine the mediation model dyadically, we applied the Actor Partner Interdependence Mediation Model (APIMeM).

**Results:**

While in the presence of gratitude as a mediator, almost no significant direct effects from dyadic coping on relationship satisfaction were found, evidence pointed to a mediating role of gratitude within this process: Provided dyadic coping was related to higher gratitude, which was in turn related to higher own and partner relationship satisfaction. The results were similar for felt and expressed dyadic coping-related gratitude.

**Discussion:**

The finding that gratitude plays an important mediating role within the dyadic coping process offers important future directions for research as well as preventative and clinical work with couples.

## Introduction

While originally, research on gratitude mainly focused on intrapersonal benefits, such as individual well-being and psychological health (Bono et al., 2004), a growing body of evidence suggests that the effects of gratitude extend beyond intrapersonal effects. Within this literature, gratitude is understood as an emotion that arises from a recipient’s realization that a positive experience has occurred because of a benefactor’s responsive or thoughtful behavior towards one-self (Algoe, 2012). Expressed gratitude has been shown to promote interpersonal bonding (Algoe et al., 2010), intimacy (Murray and Hazelwood, 2011), commitment (Gordon et al., 2012) and relationship quality (Algoe et al., 2013). Furthermore, beneficial effects of gratitude on relationship satisfaction have been widely documented (Algoe et al., 2008, 2013; Chang et al., 2022; Park et al., 2019).

While the importance of gratitude has been supported by previous research, emphasis on specific topics, situations, or behaviors to which gratitude relates is lacking. For example, when a person says, “I experienced gratitude for my partner today” (for example, see Gordon et al., 2011), it remains unclear what exactly the person is grateful for. However, the content to which the gratitude pertains might be important since gratitude, like other emotions, can vary in its intensity (Brehm, 1999), which might depend on the nature of the stimulus that elicited gratitude – gratitude arising from a small favor like preparing a coffee for the partner may not have the same intensity as gratitude elicited by profound emotional support in times of acute need (e.g., loss of a beloved person, job loss, illness). More profound gratitude is likely to have stronger positive effects, both on the self and the partner, as well as in the short- and long-term. However, only recently, research on gratitude has started to uncover specific topics, situations, or behaviors for which the partners are grateful using qualitative data from couple interviews (Weitkamp and Roth et al., under review). Couples have described support and dyadic coping as one source of gratitude within the relationship (Weitkamp and Roth et al., under review). Dyadic coping (Bodenmann, 1995, 1997) describes the process of how partners communicate their stress, support each other during stressful times, and jointly cope with stress. Thus, dyadic coping comprises several components which are (a) stress communication, (b) supportive dyadic coping–referring to one partner supporting the other partner in their coping effort during stress, for example by giving advice or showing understanding, (c) delegated dyadic coping – referring to one partner taking over tasks or responsibilities of the other partner to reduce their stress, (d) negative dyadic coping–whereby one partner assists the other partner in an ambivalent, superficial, or hostile manner, and (e) common dyadic coping–occurring in a situation where both partners are affected by stress and engage in joint coping efforts. Dyadic coping has been repeatedly shown to be important for individual and couple well-being in various contexts such as chronic physical illness (Weitkamp et al., 2021) and cancer (Chen et al., 2021; Traa et al., 2015), mental health (Landolt et al., 2023), or child-related stressors (Roth et al., 2022). Furthermore, a meta-analysis by Falconier et al. (2015) showed that dyadic coping is highly relevant for relationship satisfaction. Recently, quantitative studies have started to examine gratitude in the context of support and dyadic coping: Experimental evidence showed that gratitude enhanced the positive effects of receiving support during a stressful situation (Deichert et al., 2021). In the context of motivation to provide support, gratitude predicted enhanced motivation to help the partner (Kindt et al., 2017). A recent study examined gratitude within the process of dyadic coping and found gratitude to partially mediate the association between dyadic coping and relationship satisfaction (Shujja et al., 2022b). These initial findings point to an explanation of the role of gratitude within the process of dyadic coping. Finally, the adaptability of gratitude in the context of relational support for personal and relational outcomes was examined (Cazzell et al., 2023). The authors found that gratitude matching the support was associated with beneficial outcomes while gratitude in excess of received support was associated with maladaptive outcomes (Cazzell et al., 2023). In sum, these studies provide cross-sectional, longitudinal, and experimental evidence of the importance of studying gratitude in the context of partner support and dyadic coping.

Both dyadic coping, as conceptualized in the Systemic-Transactional Model (STM; Bodenmann, 1997, 2005), and gratitude, whose interpersonal function is described in the find-remind-and-bind theory (Algoe, 2012) and the process model of appreciation (Gordon et al., 2012), are conceptualized as interpersonal processes. According to the STM, dyadic coping serves two main functions: a stress-related function and a relationship-related function. The stress-related function aims at preserving, restoring, and strengthening the homeostasis that has been disrupted by a stressful situation both in each partner and within the couple. The relationship-related function of dyadic coping encompasses strengthening the relationship and promoting feelings of “we-ness” (sense of belonging), mutual trust, and intimacy within the couple (Bodenmann, 1997, 2005). According to the find-remind-and-bind theory, expressed gratitude draws attention to the important social opportunity to strengthen a connection with a person who has the potential to be a high-quality relationship partner, for example, someone who offers unwavering support and enriches one’s life. Experienced or expressed gratitude thus can be helpful in *finding* a valuable partner or being *reminded* of the person’s value and *binds* partners closer together (Algoe, 2012). Besides the positive effects of experiencing or expressing gratitude, receiving gratitude plays an important role, too (Algoe, 2012; Gordon et al., 2012). The find-remind-and-bind theory assumes that gratitude motivates the benefactors to remain engaged in the relationship (Algoe, 2012). In line with this theoretical approach, Gordon et al. (2012) developed the process model of appreciation and stated that feeling appreciated by one’s partner is relevant to one’s own appreciation of the partner, which provides a sense of security and confidence and increases the maintenance of a romantic relationship.

With (at least) two persons involved in the process of gratitude and dyadic coping, different perspectives on these processes between the involved individuals are possible. Within the STM, a distinction is made between two perspectives: each person reports on one’s own dyadic coping behavior (self-level, e.g., “I show empathy and understanding to my partner”) and on the dyadic coping behavior of the partner (partner-level, e.g., “My partner shows empathy and understanding to me”). Different perspectives on gratitude are described in the Three-Factorial Interpersonal Emotions (TIE) framework (Chang et al., 2022): (a) the direction of gratitude behavior–expressing gratitude vs. receiving gratitude, (b) who observes gratitude behaviors–the acting self and the observing partner–and (c) the temporal scope of gratitude–dispositional and situational gratitude (Chang et al., 2022). A further distinction suggested in the literature is feeling vs. expressing gratitude (Gordon et al., 2011), where *felt* gratitude refers to experiencing feelings of gratitude towards another person, such as the partner, and expressed gratitude describes sharing one’s own gratitude with the other person by expressing appreciation and thankfulness (Gordon et al., 2011). Building upon the theoretical viewpoint of operant conditioning (Skinner, 1938), expressed gratitude is expected to have stronger effects due to positive reinforcement of the partner. However, while both felt and expressed gratitude were related to one’s own relationship satisfaction, surprisingly, across partners, only the partner’s felt gratitude predicted the other partner’s relationship satisfaction, whereas expressed gratitude did not (Gordon et al., 2011).

Given the interpersonal nature of both the process of dyadic coping and gratitude, both variables’ relevance for relationship satisfaction and previous research pointing to the importance of gratitude within the context of support and dyadic coping, examining these processes conjointly will further our understanding. While the findings of Shujja et al. (2022b) point to an explanation of the role of gratitude within the process of dyadic coping, further and more differentiated examinations of this process is needed since the authors did not differ between felt and expressed gratitude and did not distinguish between different perspectives (self and partner) but used an overall measure of gratitude. In addition, dyadic coping has been insufficiently differentiated by using an overall score of dyadic coping encompassing different forms (e.g., positive, negative) and different perspectives on dyadic coping (self and partner). Finally, while these initial results of a mediating role of gratitude for relationship satisfaction within the process of dyadic coping were obtained in a sample of Pakistani couples with collectivistic cultural background and Islamic religion these processes have yet to be studied in Western couples. Thus, we aimed to contribute to the line of research on gratitude within the context of dyadic coping and explore the role of gratitude in the relation between dyadic coping and relationship satisfaction in a Western European sample. Drawing upon the definition of gratitude as arising from a benefactor’s responsive or thoughtful behavior (Algoe, 2012), particularly supportive dyadic coping might elicit gratitude and the process model of appreciation (Gordon et al., 2012) furthermore states that relationship maintenance behavior in Partner A elicits gratitude in Partner B. We therefore aimed to explore if gratitude is a potential mechanism over which self-reported dyadic coping impacts both one’s own and the partner relationship satisfaction. To address the following research questions and hypotheses, we applied a dyadic approach—the Actor Partner Interdependence Model the Actor Partner Interdependence Mediation Model– to take different perspectives on gratitude into account, which enriches the understanding of the interpersonal process. In our first research question, we aimed to replicate previous studies showing a positive link between supportive dyadic coping and relationship satisfaction (see Falconier et al., 2015, for an overview). We hypothesized a positive direct actor (H1a) and partner (H1b) effect for both women and men. In our second research question, we aimed to explore the mediating role of dyadic coping-related gratitude between supportive dyadic coping and relationship satisfaction. Specifically, we hypothesized that indirect actor effects for both expressed (H2a) and felt (H2b) gratitude mediate the relation between supportive dyadic coping and relationship satisfaction (actor-actor indirect effects). Furthermore, we hypothesized to find indirect partner effects of expressed (H2c) and felt (H2d) gratitude (actor-partner indirect effects). We refrained from formulating specific hypotheses regarding equality or differences in the effects of felt and expressed gratitude.

## Method

The current study was preregistered (doi: https: 10.17605/OSF.IO/2GBWJ) and the data and analysis code are publicly available via the Open Science Framework (https://osf.io/es7z6/).

### Participants and procedure

Cross-sectional dyadic data of *N* = 163 mixed-gender couples that were collected as part of a larger couple study in Switzerland (see doi: 10.15139/S3/IUGVBK for more information on the project) were used. Couples were recruited via newspaper and radio announcements. Inclusion criteria were the following: duration of the current relationship of at least 1 year, sufficient fluency in German, and age of 18 years or older. The Ethics Committee of the Philosophical Faculty of the University of Zurich approved the study (No. 2013.1.1, No. 17.8.2; No. 19.8.13; No. 20.6.18) and all couples gave their written informed consent. Participants completed questionnaires on a variety of individual and dyadic variables. For the current study, questionnaire data on dyadic coping, dyadic coping-related gratitude, and relationship satisfaction were used (collected in 2021).

Individuals’ ages ranged between 29 and 90 years. On average, women were *M* = 54.96 years old (*SD* = 16.19 years) and men *M* = 57.16 years old (*SD* = 16.14 years). Relationship duration ranged from 11 to 70 years and was, on average, *M* = 30.73 years (*SD* = 16.48 years). Ninety percent of the couples were married, and 84% had at least one child. Most participants were Swiss (92% of women, 88% of men). The socioeconomic background of the sample was middle to high, with 36% of women and 53% of men holding a university degree.

### Measures

The following measures were collected as part of a larger battery of measurement instruments and socio-demographic data.

#### Dyadic coping

Dyadic coping was measured using the Dyadic Coping Inventory (DCI; Bodenmann, 2008). The DCI consists of 37 items designed to assess various forms of dyadic coping. It captures both one’s own (self: e.g., “I show empathy and understanding to my partner”) and the partner’s dyadic coping (partner: e.g., “My partner shows empathy and understanding to me”). The DCI demonstrates good validity and reliability (Bodenmann, 2008). For the current study, we used the supportive dyadic coping subscale at the self-level. The subscale consists of five items, which are answered on a 5-point Likert scale (1 = *very rarely* to 5 = *very often*). Higher scores on the scale indicate higher degrees of supportive dyadic coping. In the current study, reliability was acceptable for both women (Cronbach’s *α* = 0.78) and men (*α* = 0.78).

#### Dyadic coping-related gratitude

The Dyadic Coping-related Gratitude Questionnaire (DCGQ; Bodenmann, 2019) was used to measure dyadic coping-related gratitude. The DCGQ consists of 20 items assessing both one’s own as well as the partner’s felt and expressed gratitude for received emotional and practical dyadic coping. Example items are “My partner feels gratitude towards me for my emotional understanding and empathy towards them.” (partner felt gratitude for emotional dyadic coping). For the current study, we used partner expressed (4 items) and partner felt (6 items) gratitude subscales. Items are answered on a 5-point Likert scale (1 = *never/very rarely* to 5 = *very often*). Higher scores on the scale indicate higher degrees of gratitude. In the current study, reliability for felt dyadic coping-related gratitude was excellent for women (*α* = 0.94) and men (*α* = 0.90) and for expressed dyadic coping-related gratitude excellent for both women (*α* = 0.91) and men (*α* = 0.90). A slightly adapted version of the questionnaire was successfully validated by Shujja et al. (2022a) in a study with Pakistani couples.

#### Relationship satisfaction

Relationship satisfaction was assessed with the German short version of the Couples Satisfaction Index (CSI-4; Funk and Rogge, 2007). Participants responded to four global items about one’s relationship (e.g., “In general, how satisfied are you with your relationship?”) on a 6-point Likert scale (ranging from 1 to 6). Anchors differ between items with one example being 1 = *not at all* to 6 = *completely*. Higher values on the scale indicate higher relationship satisfaction. The scale was shown to have high precision and demonstrated strong validity (Funk and Rogge, 2007). In the current study, reliability was good (α =0.89) for women and excellent (α = 0.90) for men.

### Data analysis

To test our hypotheses, the actor-partner interdependence model (APIM; Kenny et al., 2006; Kenny and Ledermann, 2010) and the actor-partner interdependence mediation model (APIMeM; Ledermann et al., 2011; Ledermann and Bodenmann, 2006) was used to examine the associations between different variables for the same dyad members (actor effects) and across dyad members (partner effects) simultaneously. We used felt and expressed dyadic coping-related gratitude as mediators while controlling for relationship duration. We ran two separate models for felt and expressed dyadic coping-related gratitude due to convergence issues when including both forms of gratitude within one model. The following steps were performed separately. (1) Estimating the effects of the unconstrained model, (2) systematically introducing equality constraints by setting the actor effects, the partner effects, and both the actor and partner effects equal across dyad members. The hypothesized effects were tested for significant differences between women and men. The models were compared using the following goodness of fit criteria (Hu and Bentler, 1999): comparative fit index (CFI > 0.95 indicating good fit), the root mean square error of approximation (RMSEA <0.06 indicating good fit), the standardized root mean square residual (SRMR <0.08 indicating good fit) and Akaike information criterion (AIC) and Bayesian information criterion (BIC). Nested models were compared with the χ^2^ difference test. The analyses were performed using structural equation modeling (SEM) and the lavaan package (Rosseel, 2012) in R version 2023.06.1 + 524 (R Core Team, 2023). The statistical significance of the indirect effects was tested using bootstrapping and 5,000 bootstrap samples with bias-correction of confidence intervals (Preacher and Hayes, 2008).

## Results

### Preliminary analyses

Means and standard deviations of the study variables, as well as within- and between-person correlations, are shown in Table 1. Within- and between-person correlations were positive for all variables with small to large effect sizes and men reported significantly higher mean values than women across all study variables.

**Table 1:** Means, standard deviations, and correlations of study variables.

	Women	Men					
Variable	*M* (*SD*)	*M* (*SD*)	*t*	1.	2.	3.	4.
1. Supportive dyadic coping	3.65 (0.64)	3.78 (0.62)	−2.16^*^	0.30***	0.51***	0.44***	0.39***
2. Felt gratitude	3.55 (0.91)	3.74 (0.63)	−2.64^**^	0.44***	0.33***	0.79***	0.58***
3. Expressed gratitude	3.23 (0.94)	3.54 (0.76)	−3.99^***^	0.32***	0.73***	0.29***	0.63***
4. Relationship satisfaction	4.9 (0.91)	5.11 (0.71)	−3.40^***^	0.32***	0.36***	0.41***	0.58***

Values below the diagonal are for men, those above the diagonal for women. Values on the diagonal in bold are between-partner correlations. *t* = *t*-value from paired *t*-test comparing women and men. *p ≤ 0.05, **p ≤ 0.01, ***p ≤ 0.001.

### APIM without mediation

We started by fitting an actor-partner interdependence model without mediator (APIM) to test the direct effect of dyadic coping on relationship satisfaction. Results on model comparison and fit indices are shown in Table 2. Among the nested model, the APIM with equal actor and partner effects across dyad members showed the best fit with the data. The results from this model (see Table 3) revealed significant actor but not partner effects thereby confirming H1a but not H1b. Results on the unconstrained APIM are shown in the Supplementary Table S1.

**Table 2:** Fit indices for model comparison of constrained actor-partner interdependence models.

Model	χ^2^			Δχ^2^			RMSEA	SRMR	CFI	AIC	BIC
	Value	*df*	*p*	ΔValue	Δ*df*	Δ*p*					
Comparison 1											
Unconstrained	0.0	0	–	–	–	–	–	–	1.000	2641.1	2703.0
Equal actor effects	2.05	1	0.153	2.05	1	0.0153	0.080	0.025	0.992	2641.2	2699.9
Equal actor and partner effects	2.87	2	0.364	0.82	1	0.211	0.000	0.028	0.993	2640.0	2695.7
Comparison 2											
Unconstrained	0.0	0	–	–	–	–	–	–	1.000	2641.1	2703.0
Equal partner effects	0.17	1	0.676	0.17	1	0.676	0.000	0.007	1.000	2639.3	2698.1
Equal actor and partner effects	2.87	2	0.100	2.69	1	0.576	0.052	0.028	0.993	2640.0	2695.7

Relationship duration was entered as a control variable. Significant *p*-values (≤ 0.05) are shown in bold. RMSEA, root mean square error of approximation; SRMR, standardized root mean square residual; CFI, comparative fit index; AIC, Akaike information criterion; BIC, Bayesian information criterion.

**Table 3:** Model results of the constrained actor-partner interdependence model.

Effect	Standardized estimate	*SE*	*p*	95% CI
Actor effects				
SDC_F_ 🡒 RS_F_	0.383	0.061	**0.000**	[0.263, 0.503]
SDC_M_ 🡒 RS_M_	0.383	0.061	**0.000**	[0.383, 0.327]
Partner effects				
SDC_M_ 🡒 RS_F_	0.111	0.060	0.064	[0.111, 0.099]
SDC_F_ 🡒 RS_M_	0.111	0.060	0.064	[0.111, 0.077]

Relationship duration was entered as a control variable. Significant *p*-values (≤0.05) are shown in bold. SE, standard error; CI, confidence interval; SDC, supportive dyadic coping; RS, relationship satisfaction; F, female partner; M, male partner.

### Felt dyadic coping-related gratitude as mediator

**Figure 1:**
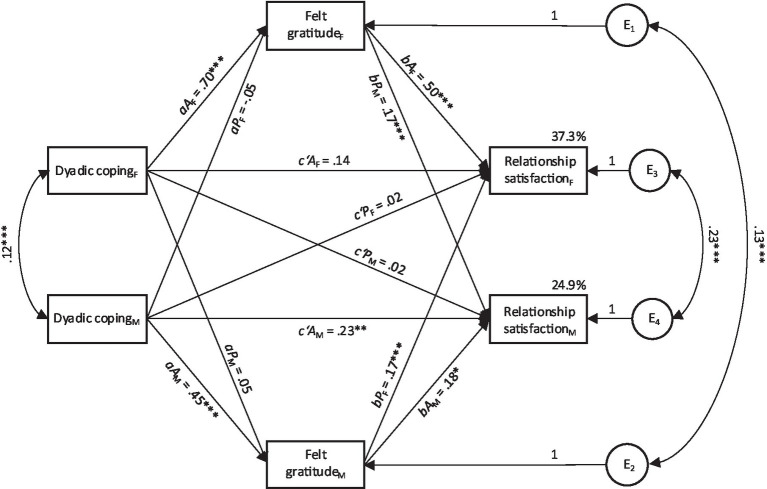
The constrained actor-partner interdependence mediation model with felt dyadic coping-related gratitude as mediator. Relationship duration was entered as a control variable. Coefficients represent standardized estimates. Single-headed arrows represent direct effects, double-headed arrows represent covariances. F, female partner; M, male partner; A, actor; P, partner; E1 to E4, residuals. *p ≤ 0.05, **p ≤ 0.01, ***p ≤ 0.001.

In the following, we will start by presenting findings from the APIMeM with felt dyadic coping-related gratitude as a mediator. Results on model comparison and model fit indices are shown in Table 4. Among the nested models, the APIMeM with equal partner effects across dyad members showed the best fit with the data. The constrained model explained 37.3% of the variance in relationship satisfaction among female partners and 24.9% among male partners. Parameter estimates of this model are provided in Table 5 and a visual representation of the model with standardized estimates is presented in Figure 1. Parameter estimates of the unconstrained model are found in the Supplementary Table S2. In the following, results from the constrained model are reported.

**Table 4:** Fit indices for model comparison of constrained actor-partner interdependence mediation models with felt dyadic coping-related gratitude as mediator.

Model	χ^2^			Δχ^2^			RMSEA	SRMR	CFI	AIC	BIC
	Value	*df*	*p*	ΔValue	Δ*df*	Δ*p*					
Comparison 1											
Unconstrained	0.0	0	–	–	–	–	–	–	1.000	3259.9	3368.2
Equal Actor Effects	10.4	3	**0.015**	10.4	3	**0.015**	0.123	0.054	0.973	3264.4	3363.4
Equal Actor and Partner Effects	19.5	6	**0.003**	9.1	3	**0.028**	0.117	0.066	0.951	3267.4	3357.1
Comparison 2											
Unconstrained	0.0	0	–	–	–	–	–	–	1.000	3259.9	3368.2
Equal Partner Effects	1.2	3	0.758	1.2	3	0.758	0.000	0.013	1.000	3255.1	3354.1
Equal Actor and Partner Effects	19.5	6	**0.003**	18.3	3	**<0.001**	0.117	0.066	0.951	3267.4	3357.1

Relationship duration was entered as a control variable. Significant *p*-values (≤ 0.05) are shown in bold. RMSEA, root mean square error of approximation; SRMR, standardized root mean square residual; CFI, comparative fit index; AIC, Akaike information criterion; BIC, Bayesian information criterion.

**Table 5:** Results of the constrained actor-partner interdependence mediation model with felt dyadic coping-related gratitude as mediator.

Effect	Path	Label	Std. Est.	*SE*	*p*	95% CI
Actor effects						
SDC_F_ 🡒 RS_F_	*c’A*_F_	Direct Effect	0.139	0.092	0.131	[−0.045, 0.318]
	*aA*_F_ *bA_F_	Actor-Actor IE	0.353	0.070	**<0.001**	[0.233, 0.511]
	aP*bP	Partner-Partner IE	0.009	0.012	0.429	[−0.009, 0.039]
	*aA*_F_*bA_F_ + aP*bP	Total IE	0.362	0.072	**<0.001**	[0.238, 0.523]
	*aA*_F_*bA_F_ + aP*bP + *c’A*_F_	Total Effect	0.501	0.085	**<0.001**	[0.337, 0.674]
Actor effects						
SDC_M_ 🡒 RS_M_	*c’A*_M_	Direct Effect	0.226	0.095	**0.018**	[0.047, 0.292]
	*aA*_M_ *bA_M_	Actor-Actor IE	0.082	0.040	**0.040**	[0.013, 0.173]
	aP*bP	Partner-Partner IE	0.009	0.012	0.429	[−0.009, 0.039]
	*aA*_M_*bA_M_ + aP*bP	Total IE	0.091	0.041	**0.025**	[0.021, 0.184]
	*aA*_M_*bA_M_ + aP*bP + *c’A*_M_	Total Effect	0.317	0.083	**<0.001**	[0.161, 0.484]
Partner effects						
SDC_M_ 🡒 RS_F_	*c’P*	Direct Effect	0.019	0.067	0.775	[−0.111, 0.153]
	*aA*_M_ *bP	Actor-Partner IE	0.076	0.031	**0.015**	[0.023, 0.143]
	aP*bA_F_	Partner-Actor IE	0.027	0.031	0.377	[−0.029, 0.093]
	*aA*_M_*bP + aP*bA_F_	Total IE	0.103	0.046	**0.026**	[0.022, 0.205]
	*aA*_M_*bP + aP*bA_F_ + *c’P*	Total Effect	0.122	0.060	**0.043**	[0.007, 0.245]
Partner effects						
SDC_F_ 🡒 RS_M_	*c’P*	Direct Effect	0.019	0.067	0.775	[−0.111, 0.153]
	*aA*_F_ *bP	Actor-Partner IE	0.118	0.045	**0.008**	[0.035, 0.211]
	aP*bA_M_	Partner-Actor IE	0.010	0.012	0.408	[−0.007, 0.043]
	*aA*_F_*bP + aP*bA_M_	Total IE	0.128	0.047	**0.007**	[0.040, 0.225]
	*aA*_F_*bP + aP*bA_M_ + *c’P*	Total Effect	0.147	0.053	**0.005**	[0.047, 0.252]

Partner effects are set to be equal across dyad members. Relationship duration was entered as a control variable. Significant *p*-values (≤0.05) are shown in bold. Std. Est., standardized estimate; SE, standard error; CI, confidence interval; SDC, supportive dyadic coping; RS, relationship satisfaction; F, female partner; M, male partner; A, actor; P, partner; IE, indirect effect.

#### Actor effects

The direct actor effects between dyadic coping and relationship satisfaction were significant for men (*c’A_M_*) but not for women (*c’A_F_*). In line with our second hypothesis for indirect effects (H2b), we found significant actor-actor indirect effects for felt gratitude in both women (aA_F_*bA_F_) and men (aA_M_*bA_M_). This implies that one’s own dyadic coping was related to one’s own relationship satisfaction via the partner’s felt gratitude. Testing for gender differences, the results revealed that women’s actor-actor indirect effect was significantly stronger than men’s actor-actor indirect effect (*p* < 0.001).

#### Partner effects

The direct partner effects (*c’P_F;_ c’P_M_*) were not significant. That is, relationship satisfaction of one partner was not directly related to the dyadic coping reported by the other partner. The findings supported hypothesis H2d about indirect partner effects (*aA*_M_*bP; aA_F_*bP). Specifically, for both men and women, own dyadic coping (Partner A) was positively related with partner gratitude (reported by Partner A), which, in turn, was positively associated with the partner’s relationship satisfaction (Partner B). Testing for gender differences, no difference emerged for the partner-partner indirect effects (*p* = 0.060).

Summarizing the findings on the mediating role of felt gratitude, constraining partner effects to be equal across males and females resulted in a good-fitting model. The findings point to a mediating role of felt gratitude between dyadic coping and relationship satisfaction in men and women and within actors (indirect actor effects) as well as partners (indirect partner effects), with stronger effects of the former.

### Expressed dyadic coping-related gratitude as mediator

**Figure 2:**
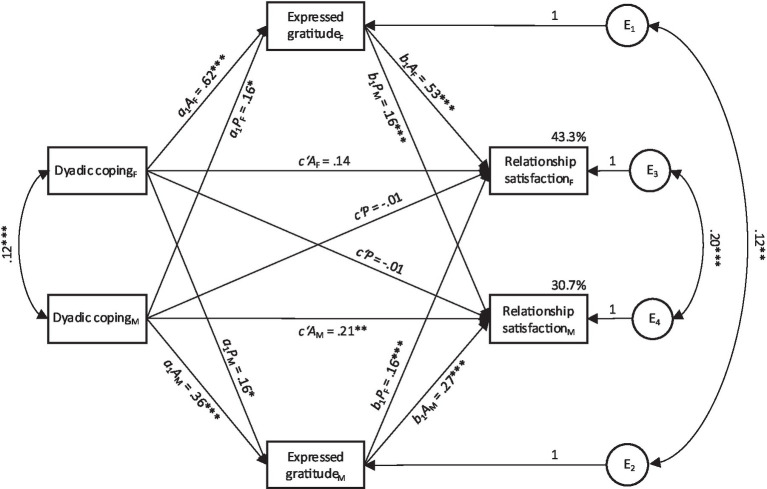
The constrained actor-partner interdependence mediation model with expressed dyadic coping-related gratitude as mediator. Relationship duration was included as a control variable. Coefficients represent standardized estimates. Single-headed arrows represent direct effects, double-headed arrows represent covariances. F = female partner; M = male partner; A = actor; P = partner; E_1_ to E_4_ = residuals. ^*^p ≤ 0.05, ^**^p ≤ 0.01, ^***^p ≤ 0.001.

Next, we present findings from the APIMeM with expressed dyadic coping-related gratitude as a mediator. Results on model comparison and model fit indices are shown in the Table 6. Again, the APIMeM with equal partner effects across dyad members yielded the best fit. The constrained model explained 43.3% of the variance in relationship satisfaction among female partners and 30.7% among male partners. Results of the constrained model are given in Table 7, and a visual representation of the model with standardized estimates is shown in Figure 2.

**Table 6:** Fit indices for model comparison of constrained actor-partner interdependence mediation models with expressed dyadic coping-related gratitude as mediator.

Model	χ^2^			Δχ^2^			RMSEA	SRMR	CFI	AIC	BIC
	Value	*df*	*p*	ΔValue	Δ*df*	Δ*p*					
Comparison 1											
Unconstrained	0.0	0	–	–	–	–	–	–	1.000	3342.6	3450.9
Equal Actor Effects	12.4	3	**0.006**	12.4	3	**0.006**	0.139	0.056	0.964	3349.0	3448.0
Equal Actor and Partner Effects	17.4	6	**0.008**	5.0	3	0.174	0.108	0.066	0.956	3348.0	3437.7
Comparison 2											
Unconstrained	0.0	0	–	–	–	–	–	–	1.000	3342.6	3450.9
Equal Partner Effects	0.3	3	0.958	0.3	3	0.958	0.000	0.008	1.000	3336.9	3435.9
Equal Actor and Partner Effects	17.4	6	**0.008**	17.1	3	**<0.001**	0.108	0.066	0.956	3348.0	3437.7

Relationship duration was entered as a control variable. Significant *p*-values (≤0.05) are shown in bold. RMSEA, root mean square error of approximation; SRMR, standardized root mean square residual; CFI, comparative fit index; AIC, Akaike information criterion; BIC, Bayesian information criterion.

**Table 7:** Results of the constrained actor-partner interdependence mediation model with expressed dyadic coping-related gratitude as mediator.

Effect	Path	Label	Std. Est.	*SE*	*p*	95% CI
Actor effects						
SDC_F_ 🡒 RS_F_	*c’A*_F_	Direct Effect	0.139	0.083	0.094	[−0.026, 0.300]
	*aA*_F_ *bA_F_	Actor-Actor IE	0.330	0.070	**<0.001**	[0.207, 0.485]
	aP*bP	Partner-Partner IE	0.027	0.013	**0.043**	[0.007, 0.062]
	*aA*_F_*bA_F_ + aP*bP	Total IE	0.356	0.073	**<0.001**	[0.234, 0.524]
	*aA*_F_*bA_F_ + aP*bP + *c’A*_F_	Total Effect	0.495	0.091	**<0.001**	[0.318, 0.677]
Actor effects						
SDC_M_ 🡒 RS_M_	*c’A*_M_	Direct Effect	0.205	0.086	**0.018**	[0.044, 0.376]
	*aA*_M_ *bA_M_	Actor-Actor IE	0.094	0.034	**0.006**	[0.042, 0.183]
	aP*bP	Partner-Partner IE	0.027	0.013	**0.043**	[0.007, 0.062]
	*aA*_M_*bA_M_ + aP*bP	Total IE	0.121	0.036	**0.001**	[0.064, 0.211]
	*aA*_M_*bA_M_ + aP*bP + *c’A*_M_	Total Effect	0.326	0.083	**<0.001**	[0.172, 0.488]
Partner effects						
SDC_M_ 🡒 RS_F_	*c’P*	Direct Effect	−0.014	0.053	0.784	[−0.118, 0.086]
	*aA*_M_ *bP	Actor-Partner IE	0.058	0.023	**0.012**	[0.023, 0.118]
	aP*bA_F_	Partner-Actor IE	0.087	0.038	**0.024**	[0.020, 0.174]
	*aA*_M_*bP + aP*bA_F_	Total IE	0.145	0.044	**0.001**	[0.069, 0.247]
	*aA*_M_*bP + aP*bA_F_ + *c’P*	Total Effect	0.130	0.059	**0.026**	[0.012, 0.241]
Partner effects						
SDC_F_ 🡒 RS_M_	*c’P*	Direct Effect	−0.014	0.053	0.784	[−0.118, 0.086]
	*aA*_F_ *bP	Actor-Partner IE	0.102	0.033	**0.002**	[0.050, 0.184]
	aP*bA_M_	Partner-Actor IE	0.043	0.021	**0.037**	[0.011, 0.095]
	*aA*_F_*bP + aP*bA_M_	Total IE	0.145	0.038	**<0.001**	[0.082, 0.234]
	*aA*_F_*bP + aP*bA_M_ + *c’P*	Total Effect	0.130	0.051	**0.011**	[0.028, 0.229]

Partner effects are set to be equal across dyad members. Relationship duration was entered as a control variable. Significant *p*-values (≤0.05) are shown in bold. Std. Est., standardized estimate; SE, standard error; CI, confidence interval; SDC, self-reported supportive dyadic coping; RS, couples relationship satisfaction; F, female partner; M, male partner; A, actor; P, partner; IE, indirect effect.

#### Actor effects

A significant direct actor effect between dyadic coping and relationship satisfaction was found in men (*c’A*_M_), indicating that their own dyadic coping had a direct positive effect on their own relationship satisfaction. In women, no significant direct actor effect was found (*c’A*_F_). In line with our hypothesis (H2a), we found significant actor-actor indirect effects for both women (a_1_A_F_*b_1_A_F_) and men (a_1_A_M_*b_1_A_M_). This implies that an increase in supportive dyadic coping (Partner A) is associated with more expressed gratitude by the partner (as reported by Partner A), which, in turn, was positively linked with one’s own relationship satisfaction (Partner A). Testing for gender differences, the results revealed that women’s actor-actor indirect effect was significantly stronger than men’s actor-actor indirect effect (*p* = 0.001).

#### Partner effects

Neither female (*c’P_F_*) nor male (*c’P_M_*) direct partner effects were significant. Thus, a person’s dyadic coping seemed to have no direct association with the partner’s relationship satisfaction. However, we found support for our hypothesis H2c in finding a significant actor-partner indirect effect for both women (*aA*_M_*bP) and men (*aA*_F_*bP). Thus, higher dyadic coping (Partner A) was associated with an elevated level of their partner’s gratitude expression (as reported by Partner A), which in turn was positively associated with Partner B’s relationship satisfaction. Testing for gender differences, no difference emerged for the partner-partner indirect effects (*p* = 0.073).

Summarizing the findings on the mediating role of expressed gratitude, constraining partner effects to be equal across males and females resulted in a good-fitting model. The findings point to a mediating role of expressed gratitude between dyadic coping and relationship satisfaction in men and women and within actors (indirect actor effects) as well as partners (indirect partner effects).

## Discussion

In the current study, we explored the role of dyadic coping-related gratitude as a potential mediating variable in the relation between supportive dyadic coping and relationship satisfaction in romantic couples using the APIM and APIMeM. In line with our hypothesis, we found direct actor effects from dyadic coping on relationship satisfaction in the APIM, whereas, contrary to our hypothesis, the direct partner effects were not significant. The significant direct actor effects are in line with previous meta-analytic findings (Falconier et al., 2015) that indicated positive effects of supportive dyadic coping on relationship satisfaction. This finding was consistently found in previous studies, even over several years (e.g., Bodenmann et al., 2006; Rusu et al., 2020). We did not find direct partner effects, indicating that one’s dyadic coping has no effect on the partner’s relationship satisfaction. This is surprising given that previous meta-analytic findings (Falconier et al., 2015) even found dyadic coping by the partner to be more relevant for relationship satisfaction that own dyadic coping. However, within-person effects were stronger than across-partner effects (Falconier et al., 2015) which might explain the non-significant partner effects in the current study.

In the APIMeM, only a few direct actor effects were significant (direct actor effect of men among felt and expressed gratitude, no direct partner effects). Since we tested for the mediating role of gratitude, the direct effects between dyadic coping and relationship satisfaction are attenuated by both partners’ gratitude and might thus fail to reach significance. These results suggest a mediating role of gratitude. Indeed and in line with our second research question, we found evidence for the importance of dyadic coping-related gratitude in the process of dyadic coping for relationship satisfaction within actors in both women and men, for felt and expressed gratitude: The effect from dyadic coping on relationship satisfaction was mediated via dyadic coping-related gratitude. Thus, for one’s own relationship satisfaction, it seems that it is not the own provision of dyadic coping that is relevant but the gratitude of the partner for the dyadic coping provision (indirect actor effects). This is in line with previous findings showing that being the recipient of a gratitude expression (perceiving gratitude in the partner for one’s effort) enhances the relationship (Gordon et al., 2012; Williams and Bartlett, 2015) and this enhances the relationship even more strongly than expressing gratitude (Chang et al., 2022; Park et al., 2019). Altogether, this points to the importance of feeling appreciated by one’s partner through receiving gratitude for one’s own dyadic coping provision. This finding also aligns with the find-remind-and-bind theory (Algoe, 2012) and the process model of appreciation (Gordon et al., 2012) both of whom emphasized the importance of receiving appreciation from the partner. Furthermore, in the context of dyadic coping, gratitude might serve as an exchange good to maintain equity between partners. According to the equity theory (Adams, 1965), a social exchange is perceived as fair if the inputs and outputs of both persons are similar. Having limited resources to provide dyadic coping when stressed, gratitude for the received dyadic coping might contribute to equity for both partners.

Besides support for our second research question within actors, we also found evidence for the relevance of gratitude across partners. These indirect partner effects indicate that partner A provides dyadic coping, which leads to higher gratitude in B as perceived by A, which then leads to higher relationship satisfaction in B and reflects the assumption of the find-remind-and-bind theory (Algoe, 2012) of gratitude being beneficial for the expressing partner as well. The current findings support this assumption even though we used a measure of partner B’s gratitude as perceived by partner A and not the actual report of partner B about his or her own gratitude, which is a more distal measure and thus potentially less likely to show significant effects (Park et al., 2019). Interestingly, however, our results are consistent with recent findings showing that accurately perceiving one’s partner’s gratitude is beneficial for the partner’s relationship satisfaction (Tissera et al., 2023).

### Felt and expressed dyadic coping-related gratitude

Although not directly comparable since we used two different models for felt and expressed gratitude, the effects in both models were similar in magnitude, and similar patterns of significant effects were found. This is particularly interesting, given the perspective used in the current study: each participant reported on their partner’s felt and expressed gratitude. One might think that expressed gratitude is easier to detect by the partner, thus having stronger effects on relationship satisfaction. However, this was not supported by the current findings and expressed and felt gratitude showed similar results. Furthermore, Gordon et al. (2011) even found cross-partner effects for felt gratitude only and not for expressed gratitude. However, they used reports on partners’ own felt gratitude and not the partners’ felt gratitude like the current study. Together, these findings shed light on the importance of felt gratitude. Still, some questions remain: How can partners detect feelings of gratitude in the other partner when it is not explicitly expressed? Do partners accurately detect felt gratitude in the other partner—that is, does it mirror the partner’s own report on felt gratitude—or is it merely important that I perceive my partner to be grateful? Indeed, the few studies that looked at how feelings of gratitude can be demonstrated without communicating it verbally found a range of other possible expressions (Chang and Algoe, 2020; Weitkamp and Roth et al., in preparation), such as showing affection (Weitkamp and Roth et al., in preparation). Furthermore, a recent study by Tissera et al. (2023) started to examine questions about gratitude perception using an accuracy and bias framework. These authors found that persons generally underestimated their partner’s gratitude, and lower perceptions of gratitude were associated with lower own and partner (for expressed but not for felt gratitude) relationship satisfaction (Tissera et al., 2023).

### Gender differences

Even though the testing of gender differences were not the focus of this study, potential gender differences are visible. For both forms of gratitude (felt and expressed), the explained variance was higher among women than men. In both models, for felt and expressed gratitude, equality constraints for partner- but not actor effects were feasible as indicated by model comparison. Given the model estimates of the unconstrained model it is not surprising that actor equality constraints did not result in a good fitting model, since indirect actor effects among women were up to more than twice as high in magnitude compared to men’s indirect actor effects, whereas the direct actor effects were higher for men compared to women. Testing for gender differences revealed that the indirect actor effects in women were significantly stronger than in men for both felt and expressed gratitude. Interestingly, only in men, a direct actor effect of dyadic coping on relationship satisfaction remained significant after entering gratitude as a mediator to the model. Across partners, effects seemed to be similar for women and men, indicated by the permissible equality constraint of partner- but not actor effects. Furthermore, the results of testing for gender differences revealed no significant differences in indirect partner effects. Overall, the higher indirect effects in women align with previous findings that showed that women were more likely to benefit from gratitude (Kashdan et al., 2009). Furthermore, women were found to feel and express more gratitude (Kashdan et al., 2009) which was also reflected in the descriptive statistics of the current study: the lower values of partner gratitude reported by women reflect this pattern of men feeling and expressing less gratitude than women. Women, however, might have felt and expressed more gratitude, which was then perceived by men, resulting in higher partner gratitude reported by men. However, the study by Kashdan et al. (2009) was not specific to couples, and to our knowledge, gender differences in gratitude in romantic couples have not yet been systematically studied and the role of gender for gratitude within the process of dyadic coping need to be investigated further.

### Limitations and future directions

The present study has several strengths, including the decent sample size of mixed-gender couples, including felt and expressed gratitude, and testing mediating mechanisms using the APIMeM. A few limitations need to be mentioned when interpreting the findings of the current study. First and foremost, we used cross-sectional data while, for the mediation model, longitudinal data would be more appropriate to capture mediation effects (Cole and Maxwell, 2003; Maxwell and Cole, 2007). However, since the variables of interest were only available cross-sectionally in the current data set, the findings of our study lay an important ground upon which future longitudinal studies can build. A further limitation of the study is that felt and expressed dyadic coping-related gratitude were examined in two different models. Due to convergence issues, probably stemming from a too small sample size, it was not feasible to include both forms of gratitude within one model. Future studies with larger samples are thus needed to analyze both forms of dyadic coping-related gratitude conjointly to directly compare the effects of felt and expressed dyadic coping-related gratitude and, thus, allow for a more differentiated understanding of their role in the process of dyadic coping. Furthermore, the partner effects that became apparent in the constrained model only should be interpreted with caution due to the sample size sensitivity of the statistical tests and the shifts in significance in partner effects from the unconstrained to the constrained model—incorporating equality constraints led to a majority of partner effects reaching significance—may be attributed to the averaging of effects and the subsequent reduction in standard errors, enhancing the precision of estimates and consequently increasing power and the likelihood of statistical significance. A further limitation pertains to the measure of dyadic coping-related gratitude. While an adapted form of the questionnaire has been validated in Pakistani couples (Shujja et al., 2022a) a thorough validation of the German version of the DCGQ is still pending and needed for future studies using the scale. Nevertheless, reliability was satisfactory in the current sample. Finally, the generalizability of the current findings is limited since the sample consisted mostly of relatively satisfied, educated, and mixed-gender couples in a stable relationship living in Switzerland with a Western European background (Henrich et al., 2010). Thus, a lack of inclusivity, as stated by Randall et al. (2022), was also present in the current study. More diverse samples in terms of level of education, sexual orientation, and cultural background are needed in future research.

Based on the current study, interesting research questions and ideas for future studies emerged, which can promote the understanding of the role of gratitude within the dyadic coping process. It would be interesting to use different perspectives of dyadic coping and dyadic coping-related gratitude than we used in the current sample. In a dyadic setting and with reports on both one’s own and the partner’s behavior, different combinations of perspectives are conceivable. For example, since Falconier et al. (2015) found that not the own dyadic coping but dyadic coping received from the partner is more relevant to one’s own relationship satisfaction, it would be interesting to examine the role of gratitude within this process. Furthermore, different perspectives can also be adopted on gratitude: What role does own felt and expressed dyadic coping-related gratitude (as compared to the subscale of partner gratitude used in the current study) play in relationship satisfaction? Furthermore, questions about consent or dissent of these different perspectives in dyadic coping and gratitude and their implications for relationship satisfaction might be addressed. Additionally, the (non-) existence of gender differences in the role of gratitude in the process of dyadic coping in romantic couples should be examined more systematically and theory-driven in future studies. Finally, given the importance of felt gratitude as shown by the current research, questions about how (accurately) partners can detect feelings of gratitude in the other partner should be addressed in future studies.

### Practical implications

A deeper understanding of the role of gratitude within the dyadic coping process might contribute to improved designs of couple intervention programs in the future that help couples to better get through adversity and stress and maintain relationship well-being. A recently published study on a gratitude-oriented intervention showed positive effects of being aware of beneficiation, noted in a gratitude diary (comparable to felt gratitude) and the expression of gratitude towards the partner on relationship satisfaction (Leong et al., 2020). However, an important variable was sincerity or authenticity of gratitude, especially in men’s perception of their partner’s gratitude. Thus, in couple therapy, it could be an important approach to promote mutual gratitude in couples, to address the possibilities of perception, feeling, and expression, and to discuss criteria for when gratitude is perceived as beneficial (authenticity, reciprocity, matching gratitude to the support received). In addition to gratitude in relation to daily dyadic coping, an important aspect could be to recall particularly relevant stressful episodes or specific situations in which gratitude was felt towards the partner, like a key experience, and to share this with each other. Mainly, in positive psychology interventions, gratitude plays an important role and seems to be effective (see Davis et al., 2016 for a meta-analysis). Based on our findings and already existing interventions on gratitude, we are convinced that fruitful tools can be developed for couples that have the potential to further increase the efficacy of interventions aiming to improve dyadic coping and relationship functioning (e.g., Couples Coping Enhancement Training, CCET; Bodenmann and Shantinath, 2004).

## Conclusion

Our study sheds light on the importance of gratitude in the realm of dyadic coping, emphasizing its relevant role within the dyadic coping process. Although gratitude has been investigated for some time, only recent research has begun to examine gratitude within the context of support and dyadic coping. Our results strengthen findings on the relevance of gratitude within this context. Specifically, we have shown that different forms of gratitude mediated the effect of dyadic coping on relationship satisfaction, within and across partners: Increased gratitude from the partner for one’s provision of dyadic coping led to higher relationship satisfaction. Our findings align with established theories such as the find-remind-and-bind theory (Algoe, 2012) and open up the idea of an equity-related function of gratitude within the context of dyadic coping: Gratitude might serve as an exchange good which re-establishes potential imbalance. Furthermore, our results highlight that both felt and expressed gratitude are relevant for relationship satisfaction, highlighting the importance of feeling appreciated by the partner.

## Data Availability

The current study was preregistered (https://doi.org/10.17605/OSF.IO/2GBWJ) and the data and analysis code are publicly available via the Open Science Framework (https://osf.io/es7z6/).
