# Supplementary material for: Building happier bonds: gratitude as a mediator between dyadic coping and relationship satisfaction in romantic couples
Source: Front Psychol. 2024 Dec 13;15:1452397. doi: 10.3389/fpsyg.2024.1452397 (PMC11671809; doi:10.3389/fpsyg.2024.1452397)
Supplement: Supplementary file 1 [file Table_1.pdf]

Supplementary Material

Details about the whole longitudinal research project can be retrieved at

https://doi.org/10.15139/S3/IUGVBK

Supplementary Tables

Supplementary Table S1. Model Results of the Unconstrained Actor-Partner Interdependence Model. Relationship duration was entered as a control variable. Significant *p*-values (≤ .05) are shown in bold. *SE* = Standard error; CI = confidence interval; SDC = supportive dyadic coping; RS = relationship satisfaction; F = female partner; M = male partner.

Effect	Standardized Estimate	*SE*	*p*	95% CI
Actor Effects				
$SDC_F \rightarrow RS_F$	.519	.108	**.000**	[0.308, 0.730]
$SDC_M \rightarrow RS_M$	.306	.086	**.000**	[0.137, 0.475]
Partner Effects				
$SDC_M \rightarrow RS_F$	.090	.112	.420	[-0.128, 0.309]
$SDC_F \rightarrow RS_M$	.153	.083	.064	[-0.009, 0.314]

Supplementary Table S2. Results of the Unconstrained Actor-Partner Interdependence Mediation Model with Felt Dyadic Coping-Related Gratitude as Mediator. Relationship duration was entered as a control variable. Significant *p*-values (≤ .05) are shown in bold. Std. Est. = standardized estimate; *SE* = Standard error; CI = confidence interval; SDC = supportive dyadic coping; RS = relationship satisfaction; F = female; M = male; A = actor; P = partner; IE = indirect effect.

Effect	Path	Label	Std. Est.	*SE*	*p*	95% CI
Actor Effects						
SDC_F → RS_F	$c'A_F$	Direct Effect	.143	.108	.185	[-.090, .341]
	aA_F*bA_F	Actor-Actor IE	.355	.079	**<.001**	[.219, .534]
	aP_M*bP_F	Partner-Partner IE	.020	.020	.313	[-.007, .079
	$aA_F*bA_F + aP_M*bP_F$	Total IE	.376	.084	**<.001**	[.230, .568]
	$aA_F*bA_F + aP_M*bP_F + c'A_F$	Total Effect	.519	.101	**<.001**	[.319, .718]
Actor Effects						
SDC_M → RS_M	$c'A_M$	Direct Effect	.219	.119	.065	[-.016, .445]
	aA_M*bA_M	Actor-Actor IE	.090	.045	**.046**	[.018, .203]
	aP_F*bP_M	Partner-Partner IE	-.003	.016	.833	[-.042, .026]
	$aA_M*bA_M + aP_F*bP_M$	Total IE	.087	.051	.085	[.004, .207]
	$aA_M*bA_M + aP_F*bP_M + c'A_M$	Total Effect	.306	.110	**.005**	[.094, .518]
Partner Effects						
SDC_M → RS_F	$c'P_F$	Direct Effect	.006	.114	.959	[-.209, .233]
	aA_M*bP_F	Actor-Partner IE	.095	.058	.098	[-.005, .229]
	aP_F*bA_F	Partner-Actor IE	-.011	.047	.814	[-.102, .086]
	$aA_M*bP_F + aP_F*bA_F$	Total IE	.084	.081	.298	[-.064, .260]
	$aA_M*bP_F + aP_F*bA_F + c'P_F$	Total Effect	.090	.109	.409	[-.112, .313]
Partner Effects						
SDC_F → RS_M	$c'P_M$	Direct Effect	.025	.091	.782	[-.148, .209]
	aA_F*bP_M	Actor-Partner IE	.107	.056	.055	[-.002, .219]
	aP_M*bA_M	Partner-Actor IE	.020	.020	.313	[-.007, .079]
	$aA_F*bP_M + aP_M*bA_M$	Total IE	.127	.058	**.029**	[.021, .246]
	$aA_F*bP_M + aP_M*bA_M + c'P_M$	Total Effect	.152	.078	.051	[-.001, .305]

Supplementary Table S3. Results of the Unconstrained Actor-Partner Interdependence Mediation Model with Expressed Dyadic Coping-Related Gratitude as Mediator. Relationship duration was entered as a control variable. Significant *p*-values (≤ .05) are shown in bold. Stand. Est. = standardized estimate; *SE* = Standard error; CI = confidence interval; SDC = supportive dyadic coping; RS = relationship satisfaction; F = female; M = male; A = actor; P = partner; IE = indirect effect.

Effect	Path	Label	Std. Est.	*SE*	*p*	95% CI
Actor Effects						
$SDC_F \rightarrow RS_F$	$c'A_F$	Direct Effect	.148	.093	.110	[-.037, .328]
	aA_F*bA_F	Actor-Actor IE	.343	.077	**<.001**	[.216, .531]
	aP_M*bP_F	Partner-Partner IE	.028	.021	.186	[.000, .091]
	$aA_F*bA_F + aP_M*bP_F$	Total IE	.371	.080	**<.001**	[.238, .561]
	$aA_F*bA_F + aP_M*bP_F + c'A_F$	Total Effect	.519	.101	**<.001**	[.326, .714]
Actor Effects						
$SDC_M \rightarrow RS_M$	$c'A_M$	Direct Effect	.198	.104	**.058**	[.005, .411]
	aA_M*bA_M	Actor-Actor IE	.087	.039	**.026**	[.028, .184]
	aP_F*bP_M	Partner-Partner IE	.021	.023	.345	[-.010, .081]
	$aA_M*bA_M + aP_F*bP_M$	Total IE	.108	.046	**.020**	[.032, .217]
	$aA_M*bA_M + aP_F*bP_M + c'A_M$	Total Effect	.306	.111	**.006**	[.101, .533]
Partner Effects						
$SDC_M \rightarrow RS_F$	$c'P_F$	Direct Effect	-.027	.083	.744	[-.179, .147]
	aA_M*bP_F	Actor-Partner IE	.050	.033	.132	[.002, .137]
	aP_F*bA_F	Partner-Actor IE	.067	.064	.300	[-.049, .203]
	$aA_M*bP_F + aP_F*bA_F$	Total IE	.117	.075	.120	[-.014, .286]
	$aA_M*bP_F + aP_F*bA_F + c'P_F$	Total Effect	.090	.109	.409	[-.106, .318]
Partner Effects						
$SDC_F \rightarrow RS_M$	$c'P_M$	Direct Effect	-.005	.081	.949	[-.169, .148]
	aA_F*bP_M	Actor-Partner IE	.109	.044	**.013**	[.036, .215]
	aP_M*bA_M	Partner-Actor IE	.048	.028	.089	[.006, .123]
	$aA_F*bP_M + aP_M*bA_M$	Total IE	.158	.049	**.001**	[.076, .275]
	$aA_F*bP_M + aP_M*bA_M + c'P_M$	Total Effect	.153	.079	.053	[-.001, .314]
